# Epidemiology and Maternal-Fetal Outcomes of Pregnancy-Related Acute Kidney Injury: A Prospective Observational Study of a Predominantly Dialysis-Requiring Cohort at a Tertiary Care Center in Northern India

**DOI:** 10.7759/cureus.113964

**Published:** 2026-08-04

**Authors:** Nikhil Srivastava, Sanjay Bharati, Shivendra Singh, Shikha Sachan, Shaurya Sisodia, Abeer Kulsoom

**Affiliations:** 1 Nephrology, Institute of Medical Sciences, Banaras Hindu University, Varanasi, IND; 2 Obstetrics and Gynaecology, Institute of Medical Sciences, Banaras Hindu University, Varanasi, IND

**Keywords:** aki, fetal loss, iud, iugr, low birth weight, p-aki, pph, praki, pregnancy, sepsis

## Abstract

Background: Pregnancy-associated acute kidney injury (PRAKI) is a significant cause of maternal morbidity and mortality, particularly in low- to middle-income countries like India. The majority of patients with stage 1/2 acute kidney injury (AKI) recover without any long-term sequelae; however, the scenario changes completely when the patients are found to be dialysis-requiring. Fixed decline in glomerular filtration rate and progression to end-stage renal disease/long-term dialysis requirement can occur in the latter group of patients, posing significant health challenges and a burden to society. We tend to investigate this group of patients with short-term follow-up for renal outcomes.

Materials and methods: This is a single-center study at a tertiary care center in northern India. We prospectively studied patients with PRAKI regarding their clinical presentation and maternal and fetal outcomes, with follow-up for three months post-discharge. Sixty-six pregnant patients with a diagnosis of AKI were admitted to our institution from 2023 to 2025. A total of 58 patients were analyzed. Maternal and fetal outcomes were noted, and patients were followed up at one month and three months post-discharge. Maternal outcomes included in-hospital mortality, complete recovery (serum creatinine <1.4 mg/dL), chronic kidney disease (CKD), and dialysis dependency at three months.

Results: The mean age was 27 years, with 79% aged 20-30 years and 41% primigravida. Puerperal sepsis (62.1%) was the leading cause, followed by septic abortion (15.5%) and antepartum hemorrhage (APH) and/or postpartum hemorrhage (PPH). Anuria (64%) was the most common presentation. Cesarean section occurred in 65% of cases, and 72% of AKIs were diagnosed postpartum. Complications included disseminated intravascular coagulation (DIC) and multi-organ dysfunction syndrome (MODS) in 28 patients. Fetal outcomes showed 48.3% preterm deliveries and 20.7% intra-uterine deaths (IUD)/stillbirths. Maternal outcomes included 41.4% renal recovery by three months, 27.6% mortality (20.7% in-hospital), and 31.1% progression to CKD, with 5.2% dialysis-dependent. Biopsies (15.5% of cases) revealed patchy cortical necrosis (five patients), IgA nephropathy (three patients), and Alport syndrome (one patient). Intensive care unit (ICU) stay, higher total leukocyte count, elevated serum glutamic oxaloacetic transaminase (SGOT) levels, and prolonged prothrombin time/international normalized ratio (PT/INR) were associated with maternal mortality. Dialysis dependency at discharge predicted adverse renal outcomes at three months.

Conclusion: Dialysis-requiring PRAKI is associated with high maternal mortality, fetal morbidity, and significant progression to CKD/end-stage renal disease. Puerperal sepsis remains the commonest cause, followed by septic abortion. Early sepsis management could reduce morbidity. The study underscores the need for improved obstetric care and timely intervention in resource-limited settings to mitigate the impact of PRAKI on maternal and fetal health.

## Introduction

Pregnancy-associated acute kidney injury (PRAKI) is associated with increased maternal morbidity and mortality. Maternal mortality associated with PRAKI has been reported to range from 9% to 55% [1,2]. The incidence of PRAKI in India ranges from 4.3% to 14.5% [3,4]. Worldwide, puerperal sepsis, septic abortion, and preeclampsia remain the dominant causes, with septic abortion being especially prevalent in low- and middle-income countries (LMICs). The incidence of acute kidney injury (AKI) due to preeclampsia is increasing in developed countries, whereas developing countries continue to be burdened by sepsis- and hemorrhage-associated AKI. The requirement for dialysis in PRAKI is a dreaded complication and is associated with increased maternal morbidity. Adverse fetal outcomes commonly associated with PRAKI include preterm labor, intrauterine growth restriction (IUGR), intrauterine death (IUD), and stillbirth. Long-term adverse renal outcomes include a decline in glomerular filtration rate (GFR), with the most dreaded complication being dialysis dependency, which has been reported in approximately 5%-19% of patients [5], with higher incidences observed in cohorts that include dialysis-requiring AKI. We undertook this study to analyze the etiologies, risk factors, and outcomes of severely affected PRAKI patients admitted to the nephrology unit, the majority of whom subsequently required dialysis.

## Materials and methods

In this prospective observational study, 66 pregnant patients diagnosed with AKI were admitted to our institution between 2023 and 2025. A total of 58 patients were analyzed (N = 58), as eight patients were lost to follow-up. Records were analyzed for demographic characteristics, obstetric history, and clinical profile at admission [6].

We excluded patients with prior renal diseases, diabetes mellitus, hypertension, contracted kidney, renal transplant recipients, or chronic kidney disease (CKD). Causes for PRAKI were defined as the sole primary obstetrical event leading to AKI. Convenience sampling (non-probability) was done wherein all the patients presenting to us within a prespecified time period (2023-2025) and satisfying inclusion criteria were included.

Subjects were included in the study after providing written informed consent, and data were collected using a pre-structured questionnaire. A thorough obstetric, medical, and surgical history was obtained. Ethical approval was obtained from the Institutional Review Committee of the Institute of Medical Sciences, Banaras Hindu University (2023/EC/6760). Written informed consent was obtained from each participant before enrollment in the study. The researchers explained the details and purpose of the study to the participants. Confidentiality was maintained throughout the data collection period. All patients were followed for 12 weeks postpartum. Patients discharged home on conservative management were advised and encouraged to attend follow-up visits at the outpatient department or Nephrology Ward. Those who did not attend their scheduled follow-up visits were contacted by telephone and encouraged to return.

The objectives of the study were to evaluate clinical characteristics, fetal outcomes, in-hospital mortality, and recovery at discharge (primary objectives), as well as renal recovery, dialysis dependency, and overall mortality at three months (secondary objectives).

Complete recovery was defined as a serum creatinine level <1.4 mg/dL. CKD was defined as an estimated glomerular filtration rate (eGFR) < 60 mL/min/1.73 m^2^ (as per the Chronic Kidney Disease Epidemiology Collaboration (CKD-EPI)) persisting for 12 weeks.

Renal biopsy was performed in patients suspected of having glomerular disease or in those with unexplained AKI, staged according to the Kidney Disease: Improving Global Outcomes (KDIGO) classification. The patient information sheet and data record form are provided in the Appendices.

AKI was staged according to the KDIGO classification. Preeclampsia was defined as a blood pressure (BP) reading >140/90 mmHg, diagnosed for the first time after 20 weeks of gestation, with signs and symptoms of end-organ dysfunction. Eclampsia was defined as the presence of new-onset generalized tonic-clonic seizures in a woman with preeclampsia. Hemolysis, elevated liver enzymes, and low platelet count (HELLP) syndrome was defined by the combination of thrombocytopenia (<100 G/L), elevated liver enzyme levels (aminotransferase >70 IU/L), and hemolysis. Hypotension was defined as a mean arterial pressure <65 mmHg. Sepsis was defined according to a Sequential Organ Failure Assessment (SOFA) score >1, and multi-organ dysfunction syndrome (MODS) was defined as the concomitant dysfunction of two or more organs arising from a common etiology.

Statistical analysis

All data are presented as mean ± standard deviation. Proportions were compared using the chi-square test or Fisher's exact test. Student's t-test was used to compare the means between the two groups. The relative risk (RR) of mortality with 95% confidence intervals for different organ involvement was analyzed. The RR of mortality was calculated by comparing the patients with involvement of the particular organ versus patients without involvement of that organ. P < 0.05 was considered significant. Data were analyzed using IBM SPSS Statistics for Windows, Version 25 (Released 2017; IBM Corp., Armonk, New York, United States).

## Results

Out of 2,063 patients admitted during the study period, 66 patients (3.2%) met the inclusion criteria. Eight patients were excluded due to loss to follow-up. Thus, data from 58 patients were analyzed. The mean age of the patients was 27 years, and the majority belonged to the 20-30-year age group (46 patients, 79%). Forty-one percent of the patients (N = 24) were primigravida. Eighty-six percent of the patients (N = 50) had stage 3 AKI according to the KDIGO classification, while the remaining patients had stage 2 AKI. The majority of the patients (N = 53, 91%) required renal replacement therapy (RRT), with hemodialysis performed in all such cases. Anuria (<100 mL urine in 24 hours) was the most common presentation (N = 37, 64%), followed by oliguria (N = 17, 29%). Cesarean section was performed in 65% of pregnancies (N = 37), while vaginal delivery occurred in 20% (N = 12). AKI was diagnosed during the postpartum period in 42 patients (72%), followed by the third and second trimesters (six patients each).

The most common etiology was puerperal sepsis, accounting for 62.1% of cases (N = 36) (Table 1). Septic abortion accounted for 15.5% of cases (N = 9). Antepartum hemorrhage/postpartum hemorrhage (APH/PPH) was less frequent, contributing to 13% of cases. Preeclampsia was identified in only three patients (5.1%). One patient with preeclampsia was diagnosed with HELLP syndrome. Less common causes included mismatched blood transfusion (1.7%, N = 1) and drug-induced AKI (1.7%, N = 1).

**Table 1:** Etiological factors contributing to pregnancy-related acute kidney injury (PRAKI) in 58 patients APH/PPH: antepartum hemorrhage/postpartum hemorrhage

Etiological spectrum	Frequency	Percentage (%)
Puerperal sepsis	36	62.1
Septic abortion	9	15.5
APH/PPH	8	13.7
Preeclampsia	3	5.1
Mismatch blood transfusion	1	1.7
Drug-induced (amikacin)	1	1.7
Total	58	100.0

Complications were noted in 28 patients (Table 2). The maternal complications analyzed included disseminated intravascular coagulation (DIC), MODS, and cardiovascular complications (CVS), such as hypotension. The majority of the patients (N = 15, 54%) had a combination of two or more of these complications. MODS and DIC alone were each noted in five patients (Table 2), while hypotension alone was observed in three patients.

**Table 2:** Complications

Complications	No. of patients affected	Percentage (%)
Multi-organ dysfunction syndrome (MODS)	19	32
Disseminated intravascular coagulation (DIC)	18	31
Cardiovascular complication	10	17

Fetal outcomes were recorded as healthy term birth, preterm delivery, IUD, intrauterine growth restriction (IUGR), and stillbirth (Tables 3-4). Fetal outcomes were then compared between two AKI groups (septic versus non-septic), and the results are presented in Table 5.

**Table 3:** Fetal outcomes in pregnancies affected by acute kidney injury (AKI) IUGR: intrauterine growth restriction

Fetal outcomes	No. of infants	Percentage (%)
Healthy baby	25	43.1
Preterm delivery with IUGR	11	18.9
Septic abortion	9	15.5
Intrauterine fetal demise (IUD)	8	13.8
Stillbirth	3	5.2
Preterm delivery	2	3.4
Total	58	100.0

**Table 4:** Birth weight of fetuses in pregnancies complicated by pregnancy-related acute kidney injury (PRAKI)

Birthweight	No. of infants	Percentage (%)
500-1500 g	9	18.3
1501-2500 g	15	30.7
2501-3500 g	22	44.9
>3500 g	3	6.1
Total	49	100.0

**Table 5:** Fetal outcomes among two subgroups: septic and non-septic AKI IUGR: intrauterine growth restriction; AKI: acute kidney injury

Fetal outcomes	Septic AKI (N=45)	Non-septic AKI (N=13)
Healthy baby	21	4
Preterm delivery with IUGR	6	5
Septic abortion	9	0
Intrauterine fetal demise (IUD)	6	2
Stillbirth	2	1
Preterm delivery	1	1

Table 6 summarizes the composite outcomes of 58 female patients diagnosed with PRAKI. The outcomes analyzed include recovery at various time points (discharge, one month, and three months), hospital mortality, post-discharge mortality, and progression to CKD with/without dialysis dependency.

**Table 6:** Composite outcomes in terms of renal recovery, in-hospital mortality, and persistent renal dysfunction at three months CKD: chronic kidney disease; CKD5D: CKD stage 5 requiring dialysis; ESRD: end-stage renal disease

Composite outcome	No. of patients	Percentage (%)
Recovery at discharge	3	5.2
Recovery at one month	12	20.7
Recovery at three months	9	15.5
Hospital mortality	12	20.7
Post-discharge mortality	4	6.9
CKD	15	25.9
CKD5D (ESRD)	3	5.2
Total	58	100.0

The composite outcomes indicated a varied prognosis, with 41.4% of patients (N = 24) achieving recovery within three months (5.2% at discharge (N = 3), 20.7% at one month (N = 12), and 15.5% at three months (N = 9)); 27.6% experiencing mortality (N = 16) (20.7% in-hospital (N = 12) and 6.9% post-discharge (N = 4)); and 31.1% (N = 18) progressing to chronic renal impairment (CKD, N = 15; CKD stage 5 requiring dialysis (CKD5D), N = 3). The exact cause of post-discharge mortality could not be ascertained; however, all patients who died after discharge were dialysis dependent at the time of discharge.

Analysis of biopsy findings and outcomes

A total of nine patients underwent renal biopsy because of persistent proteinuria or active urinary sediment. Five patients had patchy cortical necrosis, while three had immunoglobulin A (IgA) nephropathy. The remaining biopsy revealed Alport syndrome on electron microscopy (EM). One patient with patchy cortical necrosis recovered completely, while three patients were diagnosed with CKD at three months, of whom one remained dialysis dependent. All patients with IgA nephropathy (IgAN) showed evidence of chronicity, with interstitial fibrosis and tubular atrophy (IFTA) ranging from 25% to 55% (Table 7).

**Table 7:** Renal biopsy findings and their outcomes at three months CGN: chronic glomerulonephritis; IgAN: immunoglobulin A nephropathy; RCN: renal cortical necrosis; CKD: chronic kidney disease; CKD5D: CKD stage 5 requiring dialysis

Biopsy diagnosis	Biopsy correlation with composite outcome				
	Complete recovery	In-hospital mortality	Post-discharge mortality	CKD	CKD5D
CGN (IgAN)	0	0	0	3	0
RCN	1	0	1	2	1
Alport syndrome	0	0	0	1	0

The total number of biopsy-confirmed cases (N = 9) represented approximately 15.5% of the cohort. The analysis of mean laboratory parameters across PRAKI outcomes revealed distinct patterns (Table 8):

**Table 8:** Comparison of mean clinical and laboratory parameters among three outcome groups (Group A: recovery, Group B: mortality, and Group C: CKD at three months) *P-values were calculated using one-way analysis of variance (ANOVA). Hb: hemoglobin; TC: total leukocyte count; LDH: lactate dehydrogenase; SGOT: serum glutamic oxaloacetic transaminase; SGPT: serum glutamic pyruvic transaminase; ALP: alkaline phosphatase; INR: international normalized ratio; PCT: procalcitonin; HD: hemodialysis; ICU: intensive care unit; CKD: chronic kidney disease

Characteristics	Comparison of mean values in three groups			
	Recovery (Group A), N = 24	Mortality (Group B), N = 16	CKD (Group C), N = 18	P-value*
	Mean (Min-Max)	Mean (Min-Max)	Mean (Min-Max)	-
Age	26.6 (20-37)	27.4 (20-42)	28.8 (22-38)	0.42
Hb (g/dL)	7.9 (4.3-14)	6.4 (2.1-9.3)	7.0 (4.4-13)	0.1
TC (x10^3^/µL)	13129 (7700-24000)	18096 (6500-39000)	14088 (4500-26000)	0.045
Creatinine (mg/dL)	7.59 (3.1-15)	7.89 (3.9-12)	8.69 (2.9-26.5)	0.68
Platelets (x10^3^/µL)	99.8 (7-260)	94.5 (13.2-268)	121 (18-297)	1
Uric acid (mg/dL)	7.8 (3.8-10.5)	6.5 (4.5-8.5)	6.7 (1-16.2)	0.7
Albumin (g/dL)	2.9 (1.9-4.0)	2.8 (2.2-4.1)	3.0 (2.3-3.7)	0.8
LDH (U/L)	1333.9 (5-8919)	1396.9 (428-6873)	1485.0 (12-4621)	1.0
Total bilirubin (mg/dL)	1.42 (0.2-11.4)	0.77 (0.2-2.60)	1.34 (0.2-7.80)	0.51
SGOT (IU/L)	157 (11-734)	334 (18-2389)	402 (13-3062)	0.4
SGPT (IU/L)	93 (11-366)	346 (13-2971)	271 (11-2790)	0.3
ALP (IU/L)	291 (118-560)	355 (169-743)	276 (70-479)	0.1
INR	1.34 (0.9-2.8)	1.86 (0.9-4.44)	1.32 (1-1.98)	0.017
PCT (µg/L)	27 (0.1-250)	52 (0.50-250)	14 (0.50-75)	0.1
D-dimer (µg/mL)	36.7 (0.7-714)	17.5 (1.2-112)	7.4 (1.3-27.6)	0.6
Total HD sessions	3.3 (0-8)	6.7 (3-15)	6.9 (0-17)	0.003
ICU stay (in days)	0	5.4 (0-19)	3.5 (0-15)	0.002
Hospital stay (in days)	14.7 (5-35)	18.8 (6-44)	26.8 (2-48)	0.007

Recovery Groups

Lower total leukocyte count (TC) (11,100-14,073), creatinine (4.52-8.52), serum glutamic oxaloacetic transaminase (SGOT) (38-270), serum glutamic pyruvic transaminase (SGPT) (38-157), international normalized ratio (INR) (1.12-1.45), and prothrombin time (PT) (14-60) were observed in the recovery groups.

Mortality Groups

Higher TC (17,286-20,525), SGOT (211-702), SGPT (190-813), INR (1.81-1.88), PT (14-65), and D-dimer (9.8-20.0) were observed in the mortality groups.

CKD/End-Stage Renal Disease (ESRD) Groups

Higher creatinine (8.24-10.92) and SGPT (340 in the CKD group) reflected persistent renal and hepatic injury, while lower TC (14,523-19,167) and INR (1.29-1.33) suggested less acute inflammation than in the mortality groups.

Laboratory parameters across the two subgroups of AKI (septic versus non-septic) were also compared (Table 9).

**Table 9:** Comparison of mean clinical parameters between the septic and non-septic AKI groups AKI: acute kidney injury; SBP: systolic blood pressure; DBP: diastolic blood pressure; Hb: hemoglobin; TC: total leukocyte count; LDH: lactate dehydrogenase; SGOT: serum glutamic oxaloacetic transaminase; SGPT: serum glutamic pyruvic transaminase; INR: international normalized ratio; PCT: procalcitonin

Parameter	Septic AKI (N = 45)	Non-septic AKI (N = 13)
	Mean	Mean
SBP (mmHg)	133	144
DBP (mmHg)	81	86
Pulse (bpm)	89	92
HB (g/dL)	7.0	7.7
TC (×10^3^/µL)	15.2	13.2
Creatinine (mg/dL)	8.18	7.43
Platelets (×10^3^/µL)	94.3	141.8
Uric acid (mg/dL)	7.4	6.2
Albumin (g/dL)	2.9	2.9
LDH (U/L)	1569.4	805.3
Total bilirubin (mg/dL)	1.33	0.83
SGOT (IU/L)	349	48
SGPT (IU/L)	266	53
INR	1.56	1.20
PCT (ug/L)	38	2
D-dimer (ug/mL)	26.0	9.4

## Discussion

With the increasing availability of medical services, improved antenatal and postnatal care, and access to medical termination of pregnancy (MTP), the incidence of PRAKI is decreasing in India. The incidence of PRAKI decreased from 38.62% in 1987 to 9% in 2008 [3,7], and to 3.2% in the present study, which included predominantly PRAKI patients requiring dialysis. However, the rate of PRAKI in India remains high compared with that in developed countries (1% to 2.8%). As noted in other series [8,9], the majority of PRAKI cases occurred during the postpartum period, frequently presenting with the abrupt cessation of urine output in an otherwise asymptomatic woman, thereby marking the onset of a catastrophic illness.

The predominance of sepsis (77%) as the etiology of PRAKI aligns with the pathophysiology of obstetric complications in low-resource settings, where hypoperfusion and systemic inflammation drive renal injury [10,11]. A landmark study by Prakash et al. in 2016 found a decreasing incidence of postabortal sepsis over three decades, with a rising incidence of puerperal sepsis and APH/PPH. Other recent studies have also identified puerperal sepsis as a common cause of PRAKI [12]. The high incidence of sepsis as a major driver of AKI in this population suggests that early interventions, such as regular screening and intravenous (IV) antibiotics, could substantially reduce morbidity. Sepsis causes MODS, which further contributes to high morbidity and mortality. Septic abortion (15.5%) highlights the risks associated with unsafe reproductive practices and remains a major problem in developing countries. Its incidence varies geographically, ranging from 12% to 50% in different parts of India [13,14]. A study from Casablanca reported that preeclampsia and eclampsia accounted for 74.5% of PRAKI cases, sepsis for 11%, and hemorrhage for 7.2%. In contrast to developed countries, puerperal and postabortal sepsis remain the most common causes of PRAKI in India and accounted for about one-third of all cases in our study. The incidence of postabortal sepsis causing PRAKI has been reported to range from as low as 9.76% to as high as 59.7% in studies from India.

All of our glomerulonephritis cases presented with elevated BP and a decline in GFR prior to delivery, and a provisional diagnosis of preeclampsia was initially made but was later revised based on renal biopsy findings. Proteinuria is a common finding in both preeclampsia and glomerulonephritis; however, persistent renal dysfunction after delivery should raise suspicion for primary renal disease rather than preeclampsia. It is also noteworthy that persistent proteinuria does not exclude preeclampsia, as it has been reported to persist for as long as 6-12 months after delivery. The incidence of preeclampsia (3.4%) was low in our cohort compared with previous studies, presumably because many patients recovered after delivery in the obstetrics ward. Preeclampsia uncommonly causes AKI [15], although the incidence is higher in patients with HELLP syndrome [16]. One patient with HELLP syndrome in our cohort developed severe dialysis-requiring AKI and contributed to mortality. The rare cases of mismatched blood transfusion and drug-induced PRAKI underscore the importance of iatrogenic risks.

The mortality rate observed in our series (>20%) is higher than those found in contemporary series from India and other developing countries [12,14]. In contrast, developed countries report PRAKI mortality of 5-10% due to advanced critical care. A recent study [17] reported a 13.5-fold higher likelihood of inpatient mortality and a 10-fold higher likelihood of cardiovascular events with pregnancy-related AKI hospitalizations as compared to hospitalizations with no AKI. The presence of coagulopathy, sepsis with a higher total leukocyte count (TLC), intensive care unit (ICU) stay, and prolonged hospital stay was associated with adverse composite outcomes (in-hospital mortality and non-recovery of renal function at three months). The high maternal mortality in India remains a major social, economic, and medical concern. The higher mortality observed in many developing countries, including India, compared with developed countries is mainly attributable to sepsis-related deaths [18].

The recovery of renal function has been addressed in previous studies [19] to be around 80-90%, but the study involved predominantly dialysis-requiring AKI. In our study, sparing five patients, all were dialysis-requiring. The presence of dialysis dependency at discharge was the single most important factor in predicting long-term renal outcome. The 31.1% CKD progression rate exceeds global estimates (15-20%), likely because of the inclusion of more severe cases (e.g., dialysis-requiring AKI) with a high incidence of complications in our cohort. The high incidence of DIC and MODS in our study also contributed to the high mortality. Krishna et al. [20] found that 11 patients remained dialysis dependent at six months out of a cohort of 98 dialysis-requiring patients. A recent study reported that 2.4% of women with pregnancy-related AKI progressed to end-stage kidney disease (ESKD) requiring dialysis [16].

Biopsy-proven cortical necrosis was noted in five patients (8.5%), while a 2022 study by Shalaby and Shemies [21] reported cortical necrosis in 14.2% of postpartum AKI cases, which is slightly higher than the biopsy findings in the present study. Complete recovery is not uncommon in cases of patchy cortical necrosis. The high incidence of patchy cortical necrosis in post-obstetric cases represents a challenging clinical scenario. Studies have reported a very high incidence of cortical necrosis, ranging from 14% to 24%, in India [22]. However, a later study from the same center reported that the incidence of renal cortical necrosis following obstetric complications decreased significantly, from 4.7% in the 1990s to 0.5% of total AKI cases in the 2000s [23]. Postabortal AKI leading to renal cortical necrosis is rare (1.5%) in Western countries; however, it is not uncommon in developing countries [24]. The identification of IgAN and Alport syndrome on renal biopsy is novel, suggesting that underlying glomerular diseases exacerbate PRAKI severity. A recent study [11] also reported the occurrence of membranoproliferative glomerulonephritis (MPGN), membranous nephropathy (MN), IgAN, and focal segmental glomerulosclerosis (FSGS) as primary glomerular diseases in its cohort.

Table 10 summarizes the changing trend of PRAKI in this part of the country in previous studies [25,26].

**Table 10:** Comparison of previous studies on pregnancy-associated acute kidney injury (PRAKI) PE: preeclampsia; APH: antepartum hemorrhage; PPH: postpartum hemorrhage

Study	Year	No. of PRAKI cases (%)	Common etiologies
Prakash et al. [25]	1992-2002	125 (10.4)	Post-abortal sepsis (72%), PE (14%), puerperal sepsis (8%), APH/PPH (5.7%)
Prakash et al. [25]	2003-2014	69 (4.68)	Puerperal sepsis (33%), post-abortal sepsis (32%), APH/PPH (20%), PE (11%)
Godara et al. [26]	2014	57 (9.8%)	Puerperal sepsis (63.1%), PE (33.33%), post-abortion (22%), APH/(PPH) (22%)
Present study	2026	58 (3.2%)	Puerperal sepsis (62.1%), septic abortion (15%), APH/PPH (13.7%), preeclampsia (5.1%)

The major limitation of this study is the small sample size and the inclusion of dialysis-requiring PRAKI patients admitted to the nephrology unit; therefore, the true incidence is likely to be higher than that reported. However, severe PRAKI patients requiring urgent intervention are adequately represented in our cohort. This is also a prospective study and accurately assesses short-term renal outcomes (three months). The impact of high population density and low socioeconomic status on adverse outcomes in both infectious and non-infectious diseases has long been recognized, which is consistent with our cohort from northern India, a region with one of the highest population densities in the world.

## Conclusions

PRAKI is a distinct subset of AKI characterized by high mortality and variable progression to adverse renal outcomes. Puerperal sepsis remains the most common cause, followed by septic abortion, although the incidence of the latter has declined. This also provides a therapeutic window for interventions such as early antibiotic therapy and institutionalized abortion services. The presence of coagulopathy, sepsis, and ICU stay was associated with high mortality. Dialysis dependency at discharge was associated with post-discharge mortality and adverse renal outcomes at three months. Patchy cortical necrosis is a frequently encountered problem and usually progresses to CKD. Currently achieving zero mortality among preventable causes of AKI remains a distant goal and requires the implementation of further strategies, especially in developing countries.

## Appendices

Participant information sheet (PIS)

Protocol Title: Clinical Spectrum and Feto-Maternal Outcomes in Pregnancy-Related Acute Kidney Injury: A Nationwide Study

Protocol Number:

Principal Investigator:

Name of Participant:

Title: Clinical Spectrum and Feto-maternal Outcomes in Pregnancy-Related Acute Kidney Injury: A nationwide study

1.       You are invited to take part in this research study. The information in this document is meant to help you decide whether or not to take part. Please feel free to ask if you have any queries or concerns.

2.       What is your expected duration of participation?

         Your participation in this study will last for a total of 24 months.

3.       What procedures will be followed during this study?

We will collect information on your demographic and clinical profile. We will also collect data from investigations done during the evaluation and treatment.

4.       What are the risks and discomforts to you?

             There is no risk or discomfort for the patient.

5.       What benefits are expected from this research?

This is a prospective observational study to learn the spectrum and outcome of Pregnancy-Related Acute Kidney Injury.

6.       What are the alternatives available to you?

You can choose to opt out of the study.

7.       Are the data/records of the participant kept confidential?

Yes. All data will be stored pseudonymously in a confidential manner.

8.       What will be the treatment schedule(s)?

  Your treatments will be decided by your treating physician, the study will not cause any interference in the procedure.

9.       What compensation and/or treatment(s) are available to the Participant in the event of a trial-related injury?

We do not anticipate any harm on account of the study procedure. There no compensation or insurance will be provided to the patients.

10.    Whom to contact for trial related queries and what are the rights of Partcipants in the event of any injury?

For trial-related queries, patients need to contact Dr Priti Meena Study subjects have to right to opt out of the study during any point.

11.    Are the participants paid to take part in this study?

        Participants will not be paid to participate in the study.

12.    What are your responsibilities during participation in the study?

You or your representative will be notified promptly if significant new findings develop during the research, which may affect your willingness to continue participation.

13.    Your participation may be terminated by the Investigator without your consent if it is deemed appropriate by the investigator in the interest of your health or significant deviation from study procedures.

14.    Your participation in the study does not entail any direct extra expenditure from your side. You will not be provided with any remuneration for participation in the study.

15.    You can withdraw from the research and procedures at any time without giving any reasons whatsoever. You will continue to avail of medical services without any bias or prejudice.

16.    The procedure does not involve risks (to you or to the embryo or fetus, if you were to father a child or become pregnant) which are currently unforeseeable

17.    Approximately, 50-100 participants will be enrolled in the study

18.    There is no other specific information.

Contact persons:

For further information/questions, you can contact us at the following address:

Principal Investigator:

Patient Record Form

Section A

1.         Patient ID

2.         Site

3.         Has the patient provided consent for participating in the study?

4.         Age (completed years)

5.         First & Last Name

6.         City/Village

7.         Education

8.         Booked or unbooked pregnancy

9.         Employment

10.       Monthly family income

11.       Past history of kidney disease

12.       Past history of thrombocytopenia, anaemia, blood transfusion

13.       Past history of PIH, pregnancy loss

14.       Other Relevant past histories

15.       Family history of kidney disease

16.       CVD/hypertension/Diabetes

Section B:

Current details of pregnancy

1.         Gravida

2.         Para

3.         Hypertension

B(P Max (Sys, Dia),

No. of anti-hypertensives, any HTN urgency, LVH, retinopathy)

4.         Date of delivery

5.         Home or hospital delivery

6.         Timing of delivery (gestation week)

7.         Vaginal / lower (uterine) segment Caesarean section (LSCS)

8.         Indication of LSCS

9.         Post-partum haemorrhage (Y/N)

10.       Any placental abnormality

11.       History of blood transfusion (BT)

12.       Details of Blood transfusion (type and quantity)

13.       Presence of preeclampsia/eclampsia/HELLP

14.       Hypotension during delivery

15.       Likely causes of hypotension (Septic/Hypovolemic/other)

Section C

Details of Acute kidney injury (AKI)

1.         Timing of AKI

2.         KDIGO staging

3.         Oliguric/anuric

4.         Maximum creatinine

5.         Dialysis requirement

6.         Modality of Dialysis

7.         Vascular access

8.         Number of sessions of dialysis

Section D: Details of biopsy if done.

1.         Timing of biopsy -in relation to onset of AKI

2.         Glomeruli

3.         Tubulointerstitial compartment

4.         Vessels

5.         IF

6.         EM

Section E: Imaging

1.         USG- Kidney Size, CMD, Echogenicity, cortical scars; CT- Cortical necrosis [complete/partial

2.         Any other

Section F:

Details of ICU stay (if required)

1.         Ventilator

2.         Duration

3.         APACHE Scoring (if ICU stay +)

4.         Ionotropic requirment

5.         Systemic anticoagulation (yes/no)

Section G: Pregnancy and renal outcomes

1.         Death 

2.         Renal recovery at discharge (complete/partial/dialysis dependent)

Section H: Fetal outcomes

1.         Preterm delivery 

2.         Stillbirth

3.         Intrauterine fetal demise

4.         Small for gestational age/IUGR

5.         Admission to neonatal ICU

6.         Birth weight

Section I: Specific treatment

1.         Antihypertensive (drugs used)

2.         Steroids

3.         Cyclophosphamide

4.         CNI

5.         Rituximab

6.         Eculizumab

Section J: Other Complications (with details)

•           Disseminated intravascular coagulation.

•           Multiple Organ Dysfunction Syndrome (MODS)

•           Thromboembolic

•           Cardiovascular

•           Infectious

Section I

Details of any other procedure done? PLEX

Lab

**Table 11:** Laboratory parameter

Test	Result
Hb	
TLC	
Platelet count	
Haptoglobulin	
Albumin	
LDH	
% Schistocytes	
Retic count	
Creatinine	
ESR	
CRP	
T Bili/Indirect B	
SGOT	
ALP	
SGPT	

**Table 12:** Laboratory parameter

Uric acid	
PT	
INR	
aPTTK/INR	
APLA	
C3 levels	
C4 levels	
ANA	
Urine protein (24 hour or UPCR)	
Urine cytology	
HIV	
HBsAg	
HCV	
Blood/Urine culture	
Procalcitonin	
ECHO Report	
Anti CFH antibody	
CFHR deletion mutation (by MLPA)	
USG	
ECHO report	
Any other relevant investigation	

Alive/Dead	
RRT on/off	
Creatinine at discharge, 30 d 90 d eGFR Urine r/m at 90 d	
Any other	
